# *Lactobacillus helveticus* SBT2171 Alleviates Perennial Allergic Rhinitis in Japanese Adults by Suppressing Eosinophils: A Randomized, Double-Blind, Placebo-Controlled Study

**DOI:** 10.3390/nu12123620

**Published:** 2020-11-25

**Authors:** Maya Yamashita, Masaya Miyoshi, Masayuki Iwai, Ryuji Takeda, Takahiro Ono, Toshihide Kabuki

**Affiliations:** 1 Milk Science Research Institute, MEGMILK SNOW BRAND Co. Ltd., 1-1-2, Minamidai, Kawagoe, Saitama 350-1165, Japan; maya-yamashita@meg-snow.com (M.Y.); m-miyoshi@meg-snow.com (M.M.); masayuki-iwai@meg-snow.com (M.I.); 2 Department of Nutritional Sciences for Well-Being, Faculty of Health Sciences for Welfare, Kansai University of Welfare Sciences, 3-11-1, Ashigaoka, Kashiwara, Osaka 582-0026, Japan; rtakeda@tamateyama.ac.jp; 3 Ueno-Asagao Clinic, 6F Kairaku Building, 2-7-5, Higashiueno, Taito, Tokyo 110-0015, Japan; t.ono@ueno-asagao.clinic

**Keywords:** *Lactobacillus helveticus* SBT2171, perennial allergic rhinitis, eosinophil, fermented milk, Japanese guidelines for allergic rhinitis

## Abstract

This article examines the effects of fermented milk (FM) containing *Lactobacillus helveticus* SBT2171 (LH2171) on the subjective symptoms of individuals with mild and moderate perennial allergy. Two hundred subjects were divided into two groups and consumed FM containing LH2171 or placebo FM once per day for 16 weeks. The primary endpoints were defined as per the degree of nasal and ocular symptoms and difficulty in daily life as determined by the Japanese guidelines for allergy rhinitis and the Japanese allergic rhinitis standard quality of life questionnaire, respectively. The secondary endpoints included parameters related to allergic symptoms in the blood and nasal fluids, as well as the mental status. The severity of allergic rhinitis significantly improved in the LH2171 group compared to that in the placebo group. Additionally, the LH2171 group showed a significantly lower degree of “stuffy nose” (as per the diary survey) than the placebo group. Eosinophil counts in the nasal fluids and in the blood were significantly lower in the LH2171 group compared to the placebo group. Thus, the oral administration of FM containing LH2171 cells alleviated perennial allergic rhinitis in individuals with mild and moderate symptoms, possibly via suppression of eosinophils in both the blood and nasal fluids.

## 1. Introduction

Allergic disorders are very common worldwide. They are characterized by nasal and ocular symptoms, such as rhinorrhea, sneezing, nasal obstruction, nose itching, and tearing and ocular itching [1,2]. In addition, these disorders impact the quality of life (QOL), including social life, work, and school performance, and reading ability, thereby decreasing productivity [3].

Epidemiological studies in Japan have revealed that approximately 25% of the Japanese population is affected by allergic diseases, including seasonal or perennial allergy, asthma, and atopic dermatitis [4]. One of the causes of the increase in allergic diseases is the highly airtight architectural style introduced in the 1960s, which resulted in exposure to increased levels of antigens, such as mites and house dust [5].

Allergy caused by house dust affects approximately 40% of the global population in the industrialized world. These individuals spend over 90% of their time indoors, in closed living and working environments [6]. House dust consists of plant pollen, human or animal skin cells, and hairs, textile fibers, paper fibers, minerals from the outdoor soil, burnt meteorite particles, among other components part of the local environment [7]. Mites are microscopic in nature, approximately 0.2–0.4 mm in size, and easily proliferate in the presence of skin cells or mold, particularly in moist and humid conditions [8]. Further, mites are abundant in bedding materials, as these organisms feed on skin and hair, and thus, people inhale this allergen in large volumes during sleep.

The major mechanism of the allergic response is the activation of T helper type (Th)2 cytokine production, which induces the production of antigen-specific immunoglobulin (Ig)Es by B cells. Allergic reactions are induced following repeated exposure to allergens [9,10]. Generally, the therapeutic strategy for perennial allergy involves pharmacotherapy, including the use of inhibitors of chemical mediators, anti-histaminic drugs, and topical steroids; however, their potential side effects over long-term use have raised concerns [11,12]. In recent years, new therapeutic modalities, such as omalizumab, a humanized anti-human IgE monoclonal antibody preparation that inhibits the binding of IgE to mast cells, have emerged in Japan [13]. Dupilumab binds to the alpha subunit of the interleukin (IL)4 receptor (IL-4Rα) and acts as a receptor antagonist to regulate the signaling of both the IL-4 and IL-13 pathways. Dupilumab received global approval for use in treating adult patients with moderate-to-severe atopic dermatitis, asthma, and nasal polyposis, whose disease is not adequately controlled with topical prescription therapies or when those therapies are not advisable [14]. However, the use of these new medicines may create a financial health care burden, because they are quite costly.

Due to the lack of approaches that can completely cure allergies, the elimination of the causative agents, such as pollen, ticks, and house dust, combined with symptomatic therapy, are recommended to alleviate allergic symptoms. As an alternative strategy, many studies have focused on food materials that may exert anti-allergic effects [15,16]. These substances can be easily consumed in the context of daily life to prevent the worsening of symptoms, and there is little concern regarding the side effects, due to long-term intake. Functional foods or supplements that alleviate allergic symptoms, have emerged in the market, and their ingredients include methylated catechins [17], polyphenols [18], bifidobacteria [19,20], and lactic acid bacteria [15,21,22,23].

To develop foods that contribute to the maintenance and promotion of health, it is important to obtain efficacy data targeting appropriate subjects and ensure appropriate labeling. Importantly, due to the large number of people suffering from seasonal and perennial allergic symptoms in Japan, the consumer affairs agency has released guidelines for the design of clinical trials on food materials [24].

*Lactobacillus helveticus* is one of the species of lactic acid bacteria, most commonly used in the production of fermented milk and some types of hard or semi-hard cheese as a starter. The versatile nature of this bacteria is based on its highly efficient proteolytic system consisting of cell-envelope proteinases and peptidases in the cytosol [25]. Recently, *Lactobacillus helveticus* SBT 2171 (LH2171) was selected as an immunomodulatory effect strain: LH2171 inhibited IL-4 and IL-13 production, while enhancing IFN-γ and IL-10 production in antigen-stimulated murine naïve splenocytes in vitro [26]. Additionally, in a murine pollen allergen-induced allergy model, LH2171 significantly decreased the pollen-specific total antibody and total IgE levels in plasma. LH2171 suppressed the proliferation and Th2 cytokine production in the submandibular lymph node cells and increased the gene expression of IL10 and Foxp3 in Peyer’s patches [27]. In addition, a randomized, double-blind, placebo-controlled study (RCT) revealed that the ingestion of fermented milk (FM) containing LH2171 daily for 12 weeks by healthy adults positive for house dust- or mite-specific antibodies improved their symptoms of ocular and nasal discomfort [28]. In contrast, ingestion of FM containing LH2171 did not affect total IgE or antigen-specific IgE levels or the concentration of Th1/Th2 cytokines in the serum [28].

Therefore, this RCT was conducted to clarify the anti-allergic mechanism of LH2171 and evaluate its effect on the QOL. The test period was extended to 16 weeks, and the Profile of Mood States 2 questionnaire (POMS2) was used to evaluate the patients’ QOL. Chemokine ligand 17 [thymus and activation-regulated chemokine (TARC)], also known as chemokine (C-C motif) ligand (CCL) 17, and eosinophils were used as blood markers, and total IgE and antigen-specific IgE titers were determined. We expect that our results will contribute to the improvement of the quality of life of individuals suffering from perennial allergies.

## 2. Materials and Methods

The CONSORT checklist is available as supporting information; see Table S1.

### 2.1. Trial Design

This was a randomized, double-blind, placebo-controlled study. Two groups of subjects were investigated, a placebo group and the LH2171 group, from July to December 2019. FM (100 g) containing LH2171 cells or a placebo FM without LH2171 cells was ingested once per day for 16 weeks. Before ingestion (0 weeks) and at 4, 8, 12, and 16 weeks after the start of the experiment, the subjects visited the Ueno Asagao clinic in Tokyo to undergo various tests. Additionally, to evaluate subjective symptoms, the subjects kept daily records on the degree of allergic symptoms, ingestion status of the test food, and usage status of any medicine (Table 1).

### 2.2. Participants

To recruit the subjects, we conducted a telephonic interview-based survey of volunteer registrants enrolled for human studies in the healthy subject bank of TES holdings (Tokyo, Japan). Screening inspection (SCR) was conducted on 381 subjects who met the inclusion and exclusion criteria (Supplementary Materials Table S2). Among those considered suitable to participate in the study by the investigator, 200 subjects were deemed eligible. The following inclusion criteria were used: (1) Japanese males and females aged 20–65 years old, (2) healthy individuals who did not have a chronic malady, (3) individuals with allergic rhinitis symptoms, (4) individuals who provided written informed consent, (5) individuals who could visit the inspection facility and be inspected on the designated days, and (6) individuals considered suitable for the study by the principal investigator. Before SCR, written informed consent was obtained from each participant.

During the study period, the principal investigator and assistants provided the subjects with the following instructions: (1) Minimize the intake of foods containing a large number of lactic acid bacteria (yogurt, drinks rich in lactic acid bacteria, kimchi, pickles, etc.); if this was unavoidable, the content and amount consumed needed to be recorded in the diary, (2) avoid irregular life activities (sleep deprivation and overeating), (3) refrain from overdosing on alcohol, (4) avoid sudden changes in drinking status, such as suddenly stopping alcohol consumption, (5) maintain the same amount and quality of food, exercise, and sleep as before the start of the study; particularly, excessive exercise or sleep deprivation on the day before the test was prohibited, (6) avoid the use of medicines (including external preparations) and Chinese herbs; if this was unavoidable, contact the consultation counter and record the name of the product used, name of the manufacturer, and reason for the use, (7) refrain from the consumption of foods with functional claims, health foods, and supplements; however if the “medicine” was used to maintain health before inclusion in the study, its continuation without changing the dosage was recommended; before any change, the individuals were instructed to contact the consultation counter and record the name of the product used, name of the manufacturer, and reason for the use, (8) there were no specific restrictions on smoking during the test period; however, individuals were advised to maintain their normal smoking habits (frequency, brand, etc.) as much as possible; sudden changes in the smoking status, such as sudden smoking cessation, were prohibited, (9) blood collection and blood donation was prohibited, other than those scheduled for the study, from enrolment to the end of this study (blood collection for medical examinations was allowed), (10) fill out the subject diary every day and submit it on the specified days, (11) record the content of meals on the survey form from 3 days before each visit inspection and submit it on the specified days, (12) visit the hospital on the prescribed inspection dates and follow the precautions for inspection, (13) participation in other human clinical trials during the study period was prohibited, and (14) other matters that may affect the test results were prohibited. Additionally, from the day before the visit inspection to the day of the visit inspection, the use of alcohol was prohibited, and the subjects were instructed to fast for 12 h before blood collection, although a small amount of water could be consumed. The Ethics Approval Code was HR-2019-YMM01.

### 2.3. Intervention

LH2171 was originally isolated by the Milk Science Research Institute, MEGMILK SNOW BRAND Co., Ltd. (Tokyo, Japan) and deposited in the International Patent Organism Depository at the National Institute of Advanced Industrial Science and Technology (Tsukuba, Ibaraki, Japan). Two types of FM were prepared: Active FM containing LH2171 and placebo FM that did not contain LH2171. The FM mixture consisted of approximately 10% skimmed milk powder, 0.8% butter with a small amount of flavor, polysaccharide as a stabilizer, and sucralose as an artificial sweetener. Active FM was fermented with starters composed of LH2171 and *Streptococcus (S.) thermophilus*. Placebo FM was fermented with *S. thermophilus* only.

Total viable lactic acid bacteria counts were obtained using the plate counting method and BCP agar plates (Nissui, Tokyo, Japan). The total cell counts of LH2171 cells were determined by quantitative PCR, as previously described [28]. Each batch of FMs was consumed within 18 days of preparation. Subjects were instructed to store the FM in a refrigerator at <10 °C to maintain its quality. Both FMs contained approximately 10^9^ colony-forming units/g of lactic acid bacteria as determined by the BCP agar plate method at the end of the consumption period. The active FM contained more than 10^7^ cells/g of LH2171 at the end of the consumption period. To ensure the quality of the samples, each FM was prepared every week during the study period. The samples were indistinguishable in taste, flavor, and appearance. The nutritional components of the two FMs were equivalent: 37 kcal, 3.0 g protein, 0.6 g fat, 4.9 g carbohydrate, and 30 mg sodium per 100 g.

### 2.4. Outcomes

Allergic symptoms and the degree of difficulty experienced in daily life were evaluated as primary outcomes. Secondary outcomes included the POMS2^®^ Japanese version and eosinophil count in the nasal discharge, total IgE, and house dust- and mite-specific IgE, and TARC levels in the blood. For background and safety evaluation, physical examination, urinalysis, hematological and biological blood testing, as well as the height, weight, body mass index, body fat percentage, temperature, systolic and diastolic blood pressures, and pulse rate were measured for each study participant. Physician interview/adverse event evaluation was performed to evaluate safety. The height was measured only at SCR to calculate the body mass index.

### 2.5. Power Analysis of Sample Size

We calculated that a sample size of 188 (94 in each group) was necessary to achieve a statistical power of 80% and α of 5% based on an effect size of 0.51, which was determined in a previous study [26] (registered number UMIN 000027791, 2018). Assuming a 5% loss in the follow-up rate, 200 participants (100 in each group) were enrolled.

### 2.6. Selection, Randomization, and Blinding

Among the 381 subjects who provided informed consent, 200 subjects were determined eligible by the physician. The subjects were randomly allocated to either the LH2171 group or the placebo group (n = 100 each) according to the stratified block randomization method based on the subjects’ sex, age, and severity classification of allergic rhinitis symptoms, as per the total score from six questions in “nasal and eye symptoms” in the Japanese allergic rhinitis standard QOL questionnaire (JRQLQ No.1). This allocation and assignment were performed by the allocation controller of Mr. Kazunori GOTO belong to TES Holdings, using a computerized random number generator, according to the provided codes. The allocation controller locked the assignment tables until the key opening day. The participants, the sponsor, investigator, the entire contract research organization staff (i.e., the director of the trial, the director of trial conduction, the person in charge of monitoring, the director of statistical analysis, and the person in charge of shipping), the medical institution staff, the IRB members, the laboratory staff and others who were involved in this trial were not involved the allocation.

### 2.7. Evaluation of Symptoms

#### 2.7.1. Severity of Allergic Rhinitis Symptoms

The severity of allergic rhinitis symptoms was classified by the physician according to the Japanese guidelines for allergy rhinitis 2016 (Revised 8th Edition) at the visit inspection [5]. Severity was evaluated on a 5-grade scale as “most severe”, “severe”, “moderate”, “mild”, and “no symptoms” based on the combination of sneezing or rhinorrhea with the strength of nasal obstruction.

#### 2.7.2. Analysis of the Treatment Effects

The treatment effects were analyzed according to the Japanese guidelines for allergy rhinitis 2016 (Revised 8th Edition) [5] and classified as worse, unchanged, improved, better, and disappearance of severe allergic rhinitis symptoms between the 0 and 16 weeks’ time-points of test food ingestion.

#### 2.7.3. Degree of Local Findings

The degree of local findings was classified by the physician according to the Japanese guidelines for allergy rhinitis 2016 (Revised 8th Edition) at the visit inspection [5]. The swelling status of the concha nasalis inferior mucosa, the color of the concha nasalis inferior mucosa, the amount of aqueous secretion, and the character of nasal mucus were evaluated on a scale of 1–4 (“+++: 4 points”, “++: 3 points”, “+: 2 points”, “−: 1 point”).

#### 2.7.4. Ocular and Nasal Symptom Survey as per the Subjects Diaries

The subjects evaluated the degree of sneezing, runny nose, stuffy nose, itchy eyes, and tearing on a daily basis on a 5-point scale: “no symptoms: 0 points”, “mild: 1 point”, “moderate: 2 points”, “severity: 3 points”, “most severe: 4 points”, from asymptomatic to the most severe. An average value was calculated each week.

#### 2.7.5. JRQLQ No.1 and POMS 2

The subjects responded to the JRQLQ No.1 [5] and POMS 2 [29,30] during the last 1–2 weeks of inspection visits.

### 2.8. Blood and Nasal Fluid Sample Analysis

The number of eosinophils in the blood was counted by microscopic observation after counting white blood cells with an automatic blood cell analyzer. [31]. The amount of TARC in the plasma was measured by the CLEIA method. The levels of house dust- or mite-specific IgE and non-specific IgE in the serum were measured by fluorescence enzyme immunoassay. The number of eosinophils in the nasal fluids was measured by the Wright staining method [32]. These measurements were performed at LSI Medience Corporation (Tokyo, Japan).

The following hematological parameters were measured in the subjects’ blood samples at the scheduled time points: White and red blood cell counts, hemoglobin, hematocrit, mean corpuscular volume, mean corpuscular hemoglobin, mean corpuscular hemoglobin concentration, and blood platelets. Total protein, creatinine, uric acid, aspartate aminotransferase, gamma-glutamyl transpeptidase, alkaline phosphatase, lactate dehydrogenase, total cholesterol, triglycerides, low-density lipoprotein cholesterol, high-density lipoprotein cholesterol, total bilirubin, glucose, and HbA1c were measured as the biochemical blood parameters at the SCR and all visit inspections; HbA1c was measured only at the SCR. Additionally, albumin, blood urea nitrogen, creatine kinase, quantified C-reactive protein, sodium, chloride, potassium, calcium, magnesium, and serum iron were measured at visits 1, 3, and 5. All measurements were performed by Hoken Kagaku, Inc. (Kanagawa, Japan).

### 2.9. Urinalysis

Urinalysis parameters, including the presence of urobilinogen, occult blood, bilirubin, ketone bodies (qualitative), glucose (qualitative), protein, pH, and urinalysis specific gravity, were measured at the scheduled time points by LSI Medience, Inc. in accordance with the global standard methods.

### 2.10. Food Frequency Questionnaire

Subjects completed a food frequency questionnaire: The breakfast, lunch, dinner, snacks, and alcohol content ingested for three days before the visit inspections were recorded. This questionnaire was used to estimate nutrient intake based on the subject’s regular diet. Energy intake, lipid content, carbohydrate content, protein mass, and dietary fiber content were calculated from these records using the HealthyMaker Pro 501 Nutrient Calculation Software (version R10; Mushroomsoft Co., Ltd., Okayama, Japan).

### 2.11. Adverse Events

A background questionnaire was completed by the subjects during the SCR period; subjective symptoms and the occurrence of adverse events during the test period were recorded in the subject’s diary. Adverse events were analyzed, and appropriate treatment was administered when needed.

### 2.12. Ethics

This study was conducted in compliance with the ethical principles of the Declaration of Helsinki and with the ethical guidelines for medical research targeting humans (MEXT, Ministry of Health, Labor, and Welfare). To ensure the safety and reliability of the test data, the study was performed based on the deliberation of the Ueno-Asagao Clinic Ethics Committee, Tokyo, Japan, on 26 June 2019 (IRB approval number: HR-2019-YMM01). Information about this study, including the study outline, methods, expected effects, side effects, and protection of privacy, was given to all subjects. Written informed consent was obtained from all subjects prior to SCR. The protocol was registered at the University Hospital Medical Information Clinical Trials Registry (UMIN: 000037329).

### 2.13. Statistics

The full analysis set population, defined as all randomly assigned subjects who consumed test food more than once during the trial period, was used for safety analysis. The per-protocol set, which was defined as subjects not matching the inclusion criteria in the full analysis set population, was used for efficiency analysis.

All data were expressed as the mean ± standard deviation. Statistical analyses were performed using the SAS statistical software, version 9.4 (SAS Institute, Cary, NC, USA) or the SPSS software, version 26 (SPSS, Inc., Chicago, IL, USA). All statistical analyses were two-tailed, with a significance level of 5%.

Differences between groups and within groups were evaluated using the Mann-Whitney *U* test or the Wilcoxon signed-rank test for score data, respectively, i.e., the severity of allergic rhinitis symptoms, degree of local findings, ocular and nasal symptom survey as per the subject’s diaries, JRQLQ No.1 and POMS 2 and eosinophils in the nasal fluids. Differences between groups and within groups were evaluated using the unpaired *t*-test or *t*-test for continuous data, respectively, i.e., blood and urinalysis data. For analyzing the treatment effect, the chi-square or the Fisher’s exact tests were used to analyze the differences between groups.

## 3. Results

### 3.1. Study Flow

**Figure 1:**
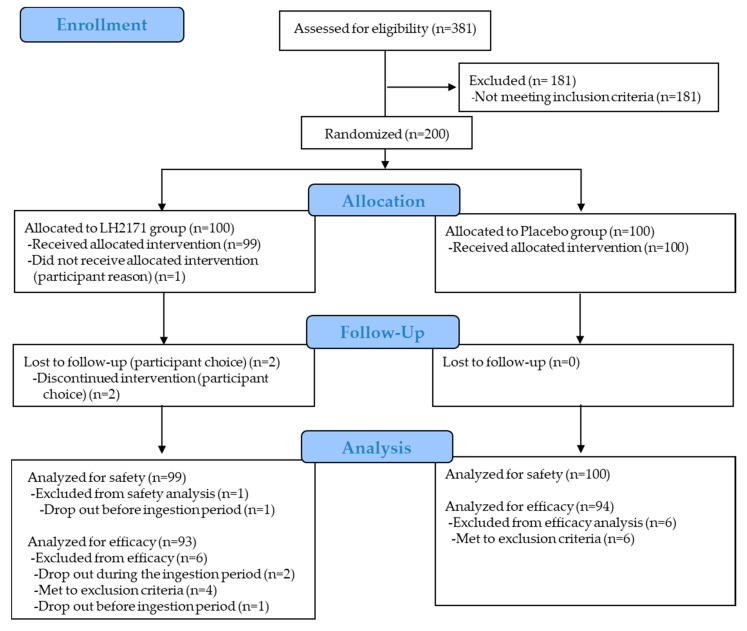
Flow diagram representing the subjects’ disposition.

Figure 1 shows the flow chart of this study. The target subjects were healthy Japanese individuals, aged more than 20 years, who experienced symptoms of nasal or ocular discomfort. Of the 381 subjects screened, 200 were deemed eligible and randomly divided into either the LH2171 group or the placebo group (n = 100 each). One female subject dropped out between the time of allocation and the 0-week time-point because she found out she was pregnant. In addition, two subjects dropped out for personal reasons during the follow-up period. Therefore, 199 subjects were included in the safety evaluation of the full analysis dataset. Efficacy was analyzed for the per-protocol data set consisting of 187 subjects (93 and 94 subjects in the LH2171 and placebo groups, respectively), excluding 10 subjects who met the exclusion criteria plus the two withdrawals for personal reasons from the full analysis dataset. Overall intake compliance rates were 99.7% and 98.0% in the placebo and LH2171 groups, respectively, according to the diaries.

### 3.2. Recruitment

Participants were recruited from July to August 2019. The trial was assigned on 14 August 2019, and the follow-up was conducted for 16 weeks, from 17 August to 10 December 2019.

### 3.3. Subjects Baseline Data

The subjects’ baseline characters are listed in Table 2. The placebo and LH2171 groups showed no differences in terms of age (LH2171 group, 45.5 ± 9.9 years old; placebo group, 44.1 ± 10.5 years old) and sex (LH2171 group: 41 males and 52 females; placebo group: 41 male and 53 female). There were no significant differences between groups regarding baseline symptoms, total scores of rhinorrhea, sneezing, nasal obstruction, nose itching, eye itching, and tearing according to the JRQLQ No.1 questionnaire and severity classification of allergic rhinitis.

### 3.4. Evaluation of Allergic Rhinitis Symptoms and Treatment Efficacy

The results of the severity of rhinitis symptoms based on the physician’s interview at the visit inspection are listed in Table 3. No significant difference was observed between the LH2171 group and placebo group with respect to the sneezing or rhinorrhea score, the nasal obstruction score, and the severity classification of allergic rhinitis symptoms score. However, in the LH2171 group, the severity classification of allergic rhinitis symptoms score significantly improved 4 and 16 weeks after the start of ingestion compared to that at the 0-week time-point.

Table 4 shows the results of the effects of the treatments on the severity of allergic rhinitis symptoms throughout the intervention. The improvement in symptoms (including improved and disappeared) was observed in 19.1% of subjects in the placebo group and 31.2% of those in the LH2171 group; therefore, a significant improvement was observed in the LH2171 group from 0 to 16 weeks (*p* = 0.024).

### 3.5. Classification of the Intranasal Evaluation by Physicians

No significant difference was observed between groups regarding the intranasal condition. In both the groups, the swelling and the color of the concha nasalis inferior significantly improved at 4, 8, 12, and 16 weeks after the start of ingestion compared to 0 weeks. In the LH2171 group, the aspect of the nasal mucus significantly improved at 4, 8, and 16 weeks after the start of ingestion, and the aqueous secretion significantly improved at 8 and 16 weeks after the start of intake compared to the 0-week time-point (Supplementary Materials Table S3).

### 3.6. Subjective Symptoms

**Figure 2:**
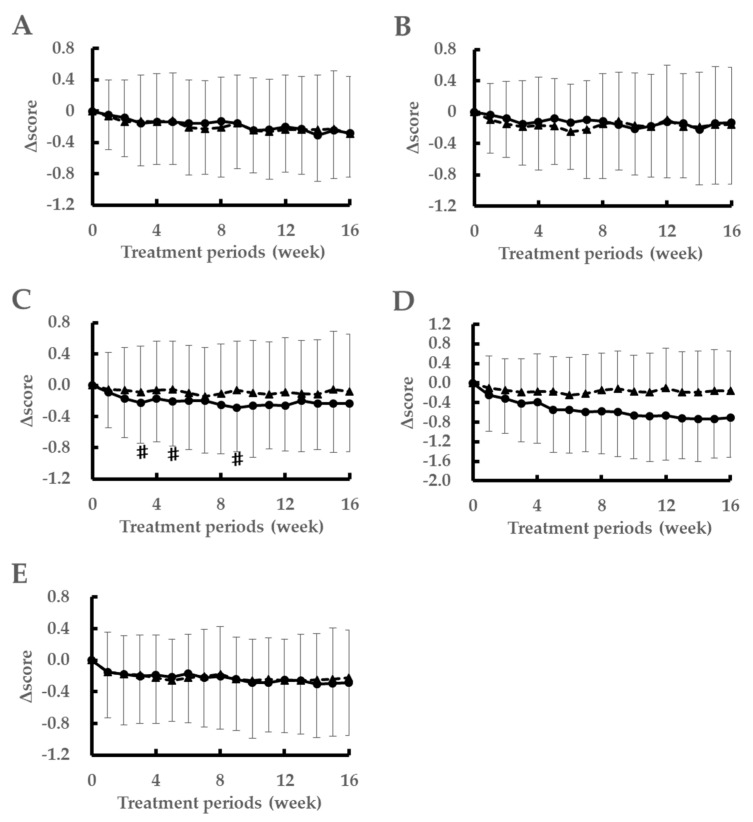
Weekly changes in the subjective symptoms scores according to the subjects’ diaries. Data are expressed as the mean ± standard deviation. ^#^ *p* < 0.05 versus the placebo group as per the Mann-Whitney *U* test. ●; LH2171 group, ▲; placebo group. (**A**) sneezing, (**B**) runny nose, (**C**) nasal blockage, (**D**) eye itching, (**E**) tearing.

Figure 2 shows the changes in the nasal or ocular symptoms’ scores according to the diaries of the subjects. The LH2171 group showed significant improvements in the stuffy nose scores at 3, 5, and 9 weeks after the initiation of ingestion compared to those in the placebo group. In addition, for the intra-group comparison of LH2171 at 16 weeks after the start of ingestion, all parameters improved significantly compared to those at the 0-week time-point.

On the contrary, in the survey of subjective symptoms as per the JRQLQ, no significant difference was found between the LH2171 and placebo groups.

Notably, this study targeted adults with mild to moderate perennial allergies, which required no medication, and evaluated whether foods supplement could be used as part of self-medication. We checked whether the subjects self-medicated to alleviate ocular or nasal allergies throughout the study period. According to the diary of the subjects, one subject took a rhinitis drug once, one subject used an eye drop for five days, one subject used an eyewash solution, and another one used an eye washer for 12 days throughout the treatment period. The other subjects did not use any medication to treat allergic symptoms.

### 3.7. Allergy-Related Immune Markers in the Blood and Nasal Fluids

**Figure 3:**
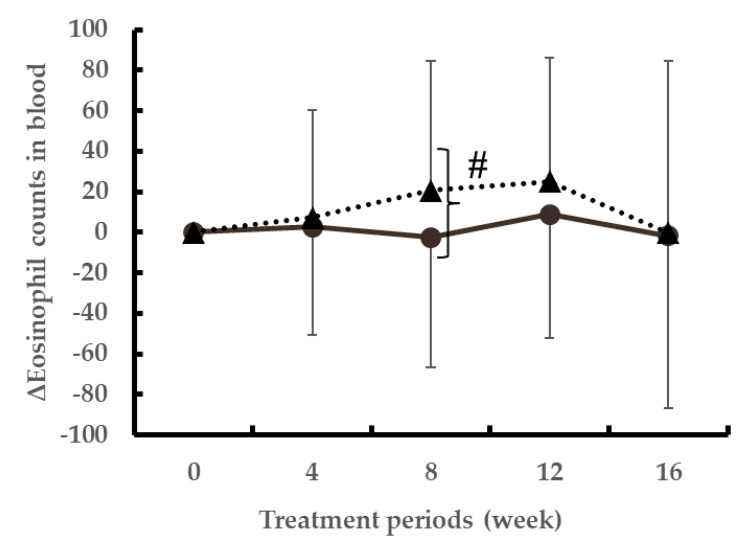
Changes in the eosinophil counts in the blood. Data are shown as the mean ± SD. Data were analyzed using the unpaired t-test. ^#^ *p* < 0.05 denotes statistically significant differences between groups. ●; LH2171 group, ▲; placebo group.

The change in the blood eosinophil counts from 0 to 8 weeks was significantly lower in the LH2171 group than in the placebo group (Figure 3). The eosinophil score in the nasal fluids was significantly lower in the LH2171 group at 4 and 12 weeks after the start of ingestion compared to that in the placebo group (Table 5). There were no significant differences in allergen-specific IgE and total IgE levels in the serum between groups (Table 5 and Table 6). The level of TARC was significantly increased in the placebo group at eight weeks after the start of ingestion compared to the value at 0 weeks, whereas in the LH2171 group, the TARC levels did not increase significantly with time (Table 6).

### 3.8. POMS2

No significant difference was observed between groups with respect to anger-hostility, confusion-bewilderment, depression-dejection, fatigue-inertia, tension-anxiety, vigorous-activity, friendliness, negative mood state, and the TMD score (Supplementary Materials Table S4).

### 3.9. Stratified Analyses

Stratified analyses were performed to assess the effects of the interventions on the pre-intake mental states, using POMS2 (Table S4). Table 7 shows the stratified JRQLQ scores with a low score for “friendliness”, showing a median of less than 51 at the 0-week time-point.

The score for eye itching significantly improved in the LH2171 group (n = 44) compared to that in the placebo group (n = 44) from 0 to 16 weeks (Table 8). In addition, concerning the severity classification of nasal symptoms, the placebo group showed improvement (improved plus disappeared) in 16.0% of subjects (6.8%), whereas LH2171 group showed improvement in 36.8% of subjects (Table 7).

### 3.10. Safety Evaluation

No adverse events were observed during the study period. Itching on the face, discomfort inside the ears, rash, common cold, trauma, and diarrhea were reported by some subjects; however, the degrees of symptoms were slight and disappeared during the study. Thus, the study physician determined that these symptoms may not be related to the food. Significant differences in some blood markers were observed between groups; however, the mean values remained within the reference ranges and were of no significant consequence, based on the physician’s judgment.

## 4. Discussion

The outcomes of this trial demonstrated that the daily intake of drinkable yogurt containing LH2171 for 16 weeks is safe and ameliorates the symptoms of nasal discomfort in subjects with perennial allergy, probably via suppressing of eosinophils.

Nasal and ocular manifestations are typical symptoms of perennial allergy. In a previous study, the administration of FM containing LH2171 cells significantly improved the nasal and ocular symptom scores in the context of perennial allergy compared with those in the placebo group [28]. However, the mechanisms underlying anti-allergic effects of LH2171 cells remained unclear, since no changes were observed in allergy-related markers, such as Th1/Th2 cytokines and IgE levels, in LH2171 versus placebo groups. Therefore, to clarify the mechanism responsible for the anti-allergic function of LH2171 cells, an increased detection power and review of parameters measured were necessary. Our results revealed that the continuous intake of DY containing LH2171 cells improved nasal discomfort caused by a perennial allergy, accompanied by a significant suppression of eosinophils in the nasal fluids and blood.

We targeted adults with mild to moderate perennial allergies, who required no medication, to determine whether food supplement can be used as part of self-medication. All but five subjects did not use medicines to treat allergic symptoms. Therefore, our subjects were considered suitable for evaluating food as part of self-medication.

Allergy is characterized by the activation of CD4+ Th2 cells and the production of Th2 associated cytokines, such as IL4, IL5, and IL13, as well as levels of IgE, which activates mast cells [33]. Activated mast cells produce histamine and release various inflammatory lipid mediators, such as prostaglandins and leukotrienes [34]. In turn, these inflammatory lipid mediators promote the migration of eosinophils into the conjunctiva and mucosa. Eosinophils regulate local immune and inflammatory responses, and their accumulation in the blood and tissue is associated with allergic symptoms [35]. Mature human eosinophils contain crystalloid secondary granules, which are primarily composed of highly charged basic proteins, including two major basic proteins, eosinophil cationic protein, eosinophil-derived neurotoxin, and eosinophil peroxidase, which cause bronchial epithelial damage and nasal obstruction [35,36]. The nasal eosinophil counts were significantly lower in the LH2171 group than those in the placebo group at 4 and 12 weeks after the start of ingestion. In addition, the non-specific IgE levels were lower in the LH2171 group than those in the placebo group at eight weeks after the start of ingestion (*p* = 0.082). Additionally, the eosinophil dynamics was significantly lower in the LH2171 group than in the placebo group at eight weeks after the start of ingestion. Since our study outcomes do not reveal whether LH2171 directly suppressed eosinophil differentiation, induction, and degranulation or indirectly affected eosinophils via the suppression of Th2 cytokines and mast cells, further studies are required in this direction.

In a previous study, the administration of LH2171 cells in an allergic mouse model suppressed the induction of IL5, a Th2 cytokine that induces eosinophils, in the lymph nodes [27]. Moreover, LH2171 cells suppressed the production of Th2 cytokines and induced the secretion of Th1 cytokines in spleen cells stimulated with antigen ex vivo [26]. We found that eosinophils were suppressed by ingestion of FM containing LH2171 cells, supporting that LH2171 cells exert anti-allergic effects by improving the Th1/Th2 cytokine balance. Further, the migration of eosinophils into the nasal mucosa induces inflammation, resulting in nasal congestion [35]. Therefore, our data suggest that the decrease of the eosinophils, due to the intake of FM containing the LH2171 cells alleviated the condition of a stuffy nose. Further studies are, however, required to determine the detailed anti-allergic mechanism of LH2171 cells.

TARC, a member of the CC chemokine family, recruits T cells and is a well-known indicator of allergic disease severity [37]. Terada et al. reported that nasal epithelial cells derived from subjects with allergic rhinitis release higher concentrations of TARC than subjects without allergic symptoms [38]. Xiao et al. proposed that the TARC blood levels while suffering from seasonal allergies are a reliable parameter to assess disease severity and monitor the response to treatment [39]. In this study, both groups showed significant decreases in blood TARC levels at 16 weeks compared to 0 weeks, suggesting that FM can alleviate perennial allergy symptoms. Moreover, considering that some of these subjects may have seasonal allergies, the improvement in the groups may be due to the time of the year the study was conducted.

Allergic diseases reduce the quality of life of patients, impacting daily life, outdoor activities, social life, sleep, body, and mental peace beyond the ocular or nasal symptoms [5,40]. In this study, the degree of improvement in mental problems were measured using POMS2. This questionnaire measures the mood scales of individuals, such as anger, depression, fatigue, tension, vigor, confusion, and friendliness, and includes 65 parameters rated on a 5-point scale [30]. No significant difference was observed between the LH2171 and placebo groups in any of the parameters, indicating that the intake of LH2171 did not affect the subjects’ mental health.

To confirm the difference in the response to allergic symptoms, due to the difference in mental status before the start of the study, a stratified analysis was performed based on the average of the subscales of POMS2. Subjects showing values below the median friendliness score indicated that the number of subjects with improvements in the classification of severity of allergic rhinitis symptoms was significantly larger in the LH2171 group than that in the placebo group. Interestingly, the classification of the severity of allergic rhinitis symptoms did not differ significantly between groups among those with friendliness scores above the median (data not shown). Moreover, in the LH2171 group, the friendliness scores below the median by POMS 2 improved significantly improved with respect to eye itching compared to the placebo group in JRQLQ No.1. Friendliness is a sub-parameter introduced in the second edition of POMS released in 2012 [29]. This scale functions as a barometer of the interpersonal domain to indicate the adaptability and quality of life of patients, as friendliness represents positive interpersonal feelings [30]. Of note, friendliness scores may be decreased by a low frequency of contact with others, modest outings, and low activity. Thus, the risk of coming into contact with allergens may also have been decreased. However, it is unclear whether LH2171 specifically affected ocular and nasal discomfort in these subjects, which should be further investigated.

Some safety parameters significantly differed between groups based on the clinical test values, but all these effects were slight and were judged by the physician to be within the physiological range. Although multiple adverse events were recorded in the diary, a causal relationship with the test food was denied by the physician. Thus, we confirmed the safety of the ingestion of FM containing LH2171 cells for 16 weeks.

## 5. Conclusions

Our study demonstrated that the daily intake of FM containing LH2171 cells by individuals suffering from perennial allergy and experiencing symptoms of ocular and nasal discomfort had no adverse effects and significantly improved nasal symptoms via suppression of eosinophils. In addition, a stratified analysis suggested that the ingestion of LH2171 improved ocular discomfort, particularly in subjects with a low friendless score before the intervention. Further studies are necessary to clarify the mechanism underlying the anti-allergic effects of LH2171.

## Figures and Tables

**Table 1:** Schedule of enrollment, intervention, and assessments.

Item	Before Allocation			Allocation	Post Allocation					Closeout
	Examination	Screening	Enrollment		Visit					
					1	2	3	4	5	
Time Point (Weeks)	−1 to −4			−1	0 W	4 W	8 W	12 W	16 W	
Enrollment:										
Eligibility screen	X		X							
Informed consent	X									
Allocation				X						
Interventions:										
Placebo group					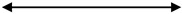					
LH2171 group					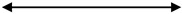					
Assessments:										
Primary outcome		X			X	X	X	X	X	X
Secondary outcome		X			X	X	X	X	X	X
Physical measurements		X			X	X	X	X	X	X
Physical examination		X			X	X	X	X	X	X
Urinalysis		X			X	X	X	X	X	X
blood test		X			X	X	X	X	X	X
Dietary survey					X	X	X	X	X	X
Dairy record					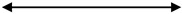					

X indicates the timing of each item.

**Table 2:** Background information on the per-protocol dataset.

Item	LH2171 Group (*n* = 93)	Placebo Group (*n* = 94)
Age (years)	45.5 ± 9.9	44.1 ± 10.5
Sex (Male/Female)	41/52	41/53
Total score of nasal and ocular symptoms ^a^	7.7 ± 3.7	7.3 ± 3.3
Severity classification of allergic rhinitis ^b^	1.6 ± 0.5	1.6 ± 0.5
Total IgE (IU/mL)	212.5 ± 491.3	268.7 ± 461.8
House dust specific IgE (IU/mL)	5.1 ± 10.5	5.6 ± 13.6
Mites specific IgE (IU/mL)	6.2 ± 13.0	6.6 ± 15.5
Cedar specific IgE (IU/mL)	13.7 ± 17.1	18.8 ± 23.2
Cypress specific IgE (IU/mL)	3.4 ± 7.5	4.5 ± 7.6
Eosinophils in blood (/μL)	174.2 ± 141.9	157.0 ± 105.8
Eosinophils in nasal fluid (score)	0.4 ± 0.7	0.5 ± 0.9

Data are presented as the mean ± SD. ^a^ The total score of nasal and ocular symptoms was calculated from the Japanese allergic rhinitis standard QOL (quality of life) questionnaire (JRQLQ No.1) questionnaire. ^b^ The severity of allergic rhinitis was classified by physicians using the subject’s diaries (sneezing, rhinorrhea, and nasal obstruction) at the SCR.

**Table 3:** Dynamic valuation of the severity of allergic rhinitis symptoms.

Items	Group	n	0 Week	4 Week	8 Week	12 Week	16 Week
Sneezing or rhinorrhea	LH2171	93	3.7 ± 0.5	3.9 ± 0.5 *	3.9 ± 0.5 **	3.8 ± 0.6	3.9 ± 0.6 *
	Placebo	94	3.8 ± 0.5	3.9 ± 0.5	3.9 ± 0.6	3.9 ± 0.5 *	3.9 ± 0.6
Nasal obstruction	LH2171	93	4.0 ± 0.6	4.1 ± 0.6	4.1 ± 0.7	4.1 ± 0.8	4.1 ± 0.7
	Placebo	94	4.1 ± 0.5	4.3 ± 0.6 **	4.2 ± 0.7 *	4.2 ± 0.7 *	4.1 ± 0.6
Classification of severity of allergic rhinitis symptoms	LH2171	93	1.4 ± 0.5	1.3 ± 0.6 *	1.3 ± 0.6	1.3 ± 0.7	1.2 ± 0.6 *
	Placebo	94	1.3 ± 0.5	1.2 ± 0.5	1.3 ± 0.6	1.2 ± 0.6	1.2 ± 0.6

Every four weeks, the severity of allergic rhinitis symptoms was classified by a physician using a 5-point scale: 0, no symptom; 1, mild; 2, moderate; 3, serve; 4, most serve. Data are expressed as the mean ± standard deviation. * *p* < 0.05, ** *p* < 0.01 compared to the 0-week time-point as per the Wilcoxon signed-rank test.

**Table 4:** The ratio of changes in the severity of allergic symptoms from 0 to 16 weeks.

Evaluation	LH2171	Placebo	*p*-Value		
Worse	14.0	11.7	0.642	^a^	
Unchanged	54.8	70.2	0.030	^a^	^#^
Improved	25.8	12.8	0.024	^a^	^#^
Better	0.0	0.0	1.000	^b^	
Disappeared	5.4	5.3	1.000	^b^	

^#^*p* < 0.05 between the group by ^a^ chi-square test or ^b^ Fisher’s exact probability test.

**Table 5:** Changes in blood eosinophils, total IgE levels, and eosinophils counts in nasal secretions.

Item	Group	n	0 Week	4 Week	8 Week	12 Week	16 Week
Nasal eosinophil count (score)	LH2171	93	0.4 ± 0.7	0.3 ± 0.7 ^#^	0.5 ± 0.9	0.4 ± 0.7 ^#^	0.3 ± 0.6
	Placebo	94	0.5 ± 0.9	0.5 ± 0.8	0.6 ± 0.9	0.6 ± 0.9	0.5 ± 0.7
Blood eosinophil count (/μL)	LH2171	93	174.2 ± 141.9	176.8 ± 141.9	170.0 ± 118.2	183.1 ± 127.3	172.6 ± 140.2
	Placebo	94	157.0 ± 105.8	164.7 ± 111.4	171.2 ± 127.4 **	180.0 ± 123.9 ***	156.7 ± 132.9
Total IgE (UA/mL)	LH2171	93	212.5 ± 491.8	204.0 ± 419.3	164.1 ± 274.9	185.8 ± 363.2	207.2 ± 468.4
	Placebo	94	268.7 ± 461.8	263.4 ± 481.4	285.6 ± 584.8	245.5 ± 484.4 *	276.5 ± 614.2

Mean ± standard deviation. ^#^ *p* < 0.05 compared with the placebo group as per the Mann-Whitney *U* test. * *p* < 0.05, ** *p* < 0.01, *** *p* < 0.001 compared with the 0-week time-point, as per the paired *t*-test.

**Table 6:** Allergy-related blood markers.

Item	Group	n	0 Week	8 Week	16 Week
House dust specific IgE (UA/mL)	LH2171	93	5.11 ± 10.54	4.98 ± 11.02	5.14 ± 10.66
	Placebo	94	5.61 ± 13.57	5.48 ± 12.38	5.94 ± 14.45
mite specific IgE (UA/mL)	LH2171	93	6.22 ± 13.02	5.90 ± 12.59	6.20 ± 13.31
	Placebo	94	6.62 ± 15.53	6.60 ± 15.46	6.94 ± 16.28
TARC (pg/mL)	LH2171	93	28.3 ± 17.4	30.3 ± 17.9	24.6 ± 15.9 *
	Placebo	94	28.2 ± 18.6	32.3 ± 23.6 **	24.9 ± 16.3 *

Mean ± standard deviation. * *p* < 0.05, ** *p* < 0.01 compared to the 0-week time-point, as per the paired *t*-test.

**Table 7:** Changes in the severity of allergic symptoms from 0 to 16 weeks in the sub-groups with a low friendliness score in POMS 2.

Evaluation	LH2171	Placebo	*p*-Value		
Worse (%)	13.6	15.9	0.76		^a^
Unchanged (%)	50.0	68.2	0.08		^a^
Improved (%)	31.8	6.8	0.006	^##^	^a^
Better (%)	0.0	0.0	1.00		^b^
Disappeared (%)	4.5	9.1	0.68		^b^

^##^*p* < 0.01 compared with the placebo group as per the ^a^ chi-square test or ^b^ Fisher’s exact probability test.

**Table 8:** Score changes in nasal and ocular symptoms as per the JRQLQ No.1 from 0 to 16 weeks in the sub-groups with a low friendliness score in Profile of Mood States 2 questionnaire (POMS 2).

Items	Group	n	4 Week	8 Week	12 Week	16 Week
Rhinorrhea	LH2171	44	0.0 ± 0.8	0.0 ± 0.7	0.1 ± 0.8	0.1 ± 0.9
	Placebo	44	0.1 ± 0.8	0.2 ± 0.7	0.0 ± 0.8	0.0 ± 0.7
Sneezing	LH2171	44	0.0 ± 0.5	0.1 ± 0.5	0.0 ± 0.6	−0.1 ± 0.7
	Placebo	44	0.0 ± 0.5	0.1 ± 0.6	0.0 ± 0.5	−0.1 ± 0.7
Nasal obstruction	LH2171	44	0.0 ± 0.4	0.1 ± 0.6	0.1 ± 0.6	0.0 ± 0.9
	Placebo	44	−0.1 ± 0.6	−0.1 ± 0.7	−0.2 ± 0.7	0.0 ± 0.6
Nose itching	LH2171	44	−0.1 ± 0.6	0.1 ± 0.6	−0.1 ± 0.7	−0.3 ± 0.6 *
	Placebo	44	0.0 ± 0.6	0.0 ± 0.8	0.0 ± 0.6	−0.2 ± 0.6 *
Eye itching	LH2171	44	−0.2 ± 0.6 *	−0.2 ± 0.9 *	−0.3 ± 0.8 *	−0.5 ± 0.8 ***^#^
	Placebo	44	−0.1 ± 0.6	0.0 ± 0.8	−0.2 ± 0.8	−0.1 ± 0.9
Tearing	LH2171	44	0.0 ± 0.5	0.0 ± 0.5	0.0 ± 0.6	0.0 ± 0.7
	Placebo	44	−0.1 ± 0.8	−0.1 ± 0.6	−0.1 ± 0.8	−0.2 ± 0.8
Nasa and ocular symptoms score (total score)	LH2171	44	−0.2 ± 2.2	0.0 ± 2.5	−0.3 ± 2.6	−0.7 ± 3.2
	Placebo	44	−0.2 ± 2.4	0.2 ± 3.0	−0.6 ± 2.8	−0.6 ± 2.9
Nasal symptoms (total score)	LH2171	44	0.0 ± 1.6	0.2 ± 1.6	0.1 ± 2.0	−0.3 ± 2.3
	Placebo	44	0.0 ± 1.5	0.2 ± 2.1	−0.2 ± 1.7	−0.3 ± 1.9
Ocular symptoms (total score)	LH2171	44	−0.2 ± 0.9	−0.2 ± 1.3	−0.3 ± 1.1	−0.5 ± 1.2 *
	Placebo	44	−0.2 ± 1.2	−0.1 ± 1.3	−0.3 ± 1.5	−0.3 ± 1.5

Mean ± standard deviation. ^#^ *p* < 0.05 compared with the placebo group as per the Mann-Whitney *U* test, * *p* < 0.05, *** *p* < 0.001 compared with the 0-week time-point, as per the Wilcoxon signed-rank test.

## Supplementary Materials

The following are available online at https://www.mdpi.com/2072-6643/12/12/3620/s1, Table S1: CONSORT 2010 checklist of information to include when reporting a randomized trial, Table S2: Exclusion criteria, Table S3: Degree classification of local findings, Table S4. POMS 2 scores indicating the psychological state (T score).
