# Supplementary material for: Lactobacillus helveticus SBT2171 Alleviates Perennial Allergic Rhinitis in Japanese Adults by Suppressing Eosinophils: A Randomized, Double-Blind, Placebo-Controlled Study
Source: Nutrients. 2020 Nov 25;12(12):3620. doi: 10.3390/nu12123620 (PMC7760906; doi:10.3390/nu12123620)
Supplement: Supplementary file 1 [file nutrients-12-03620-s001.pdf]

Table S1: CONSORT 2010 checklist of information to include when reporting a randomized trial.

Section/Topic	Item No	Checklist item	Reported on page No
Title and abstract			
	1a	Identification as a randomized trial in the title	Title
	1b	Structured summary of trial design, methods, results, and conclusions (for specific guidance see CONSORT for abstracts)	abstract
Introduction			
Background and objectives	2a	Scientific background and explanation of rationale	Introduction
	2b	Specific objectives or hypotheses	Introduction
Methods			
Trial design	3a	Description of trial design (such as parallel, factorial) including allocation ratio.	Trial design
	3b	Important changes to methods after trial commencement (such as eligibility criteria), with reasons	N.A.
Participants	4a	Eligibility criteria for participants	Participant
	4b	Settings and locations where the data were collected	Participant
Interventions	5	The interventions for each group with sufficient details to allow replication, including how and when they were actually administered	Intervention
Outcomes	6a	Completely defined pre-specified primary and secondary outcome measures, including how and when they were assessed	Outcome
	6b	Any changes to trial outcomes after the trial commenced, with reasons	N.A.
Sample size	7a	How sample size was determined	Power analysis of sample size
	7b	When applicable, explanation of any interim analyses and stopping guidelines	N.A.
Randomization:			
Sequence generation	8a	Method used to generate the random allocation sequence	Selection, randomizati on and blinding
	8b	Type of randomization; details of any restriction (such as blocking and block size)	Selection, randomizati on and blinding
Allocation concealment mechanism	9	Mechanism used to implement the random allocation sequence (such as sequentially numbered containers), describing any steps taken to conceal the sequence until interventions were assigned	Selection, randomizati on and blinding
Implementation	10	Who generated the random allocation sequence, who enrolled participants, and who assigned participants to interventions	Selection, randomizati on and blinding

Blinding	11a	If done, who was blinded after assignment to interventions (for example, participants, care providers, those assessing outcomes) and how	Selection, randomization and blinding
	11b	If relevant, description of the similarity of interventions	N.A.
Statistical methods	12a	Statistical methods used to compare groups for primary and secondary outcomes	Statistics
	12b	Methods for additional analyses, such as subgroup analyses and adjusted analyses	Statistics
Results			
Participant flow (a diagram is strongly recommended)	13a	For each group, the numbers of participants who were randomly assigned, received intended treatment, and were analyzed for the primary outcome	Study flow, Figure 1
	13b	For each group, losses and exclusions after randomization, together with reasons	Study flow, Figure 1
Recruitment	14a	Dates defining the periods of recruitment and follow-up	Recruitment
	14b	Why the trial ended or was stopped	N.A.
Baseline data	15	A table showing baseline demographic and clinical characteristics for each group	Baseline data of subjects
Numbers analyzed	16	For each group, number of participants (denominator) included in each analysis and whether the analysis was by original assigned groups	Study flow, Figure 1
Outcomes and estimation	17a	For each primary and secondary outcome, results for each group, and the estimated effect size and its precision (such as 95% confidence interval)	Results
	17b	For binary outcomes, presentation of both absolute and relative effect sizes is recommended	Results
Ancillary analyses	18	Results of any other analyses performed, including subgroup analyses and adjusted analyses, distinguishing pre-specified from exploratory	Stratified analyses
Harms	19	All important harms or unintended effects in each group (for specific guidance see CONSORT for harms)	Safety evaluation
Discussion			
Limitations	20	Trial limitations, addressing sources of potential bias, imprecision, and, if relevant, multiplicity of analyses	Discussion
Generalisability	21	Generalisability (external validity, applicability) of the trial findings	Discussion
Interpretation	22	Interpretation consistent with results, balancing benefits and harms, and considering other relevant evidence	Discussion
Other information			
Registration	23	Registration number and name of trial registry	Ethics
Protocol	24	Where the full trial protocol can be accessed, if available	Ethics
Funding	25	Sources of funding and other support (such as supply of drugs), role of funders	Conflict of interest

Table S2: Exclusion criteria.

1	Individuals who visited a hospital or use a drug to treat perennial or seasonal allergic rhinitis (except the use of nose drops and eye drops of category 3 over-the-counter drug).
2	Individuals who were treated with medicine.
3	Individuals who currently have or have a history of bronchial disease.
4	Individuals who currently have or have a history of mental disease, psychiatric disease, high blood pressure, diabetes, and hyperlipidemia.
5	Individuals who used an allergic related medicine to treat a disease in the past 1 month (except temporal usage).
6	Individuals who have food allergy to ingredients of test food.
7	Individuals who are sensitive to foods and medicines.
8	Individuals who currently have or have a history of serious hepatopathy, kidney damage, heart disease, and blood disease.
9	Individuals who currently have or have a history of endocrine disease.
10	Individuals whose BMI is over 30 kg/cm^2.
11	Individuals who donated blood over 200 mL in the past 1 month or over 400 mL in the past 3 months.
12	Individuals who have a habit of ingesting fermented milk food (over three times in a week).
13	Individuals who could not restrict their ingestion of lactic acid bacteria-rich food after providing informed consent to participate in the study.
14	Individuals who habitually ingest bifidobacteria-enriched food or food for specified health uses or foods with Function Claims to ease allergy symptoms in the past 1 month or will ingest these foods during the test period.
15	Individuals who excessively consume alcohol expressed in an amount of alcohol over 60 g/day.
16	Individuals whose life style will change during the test period.
17	Individuals who currently are pregnant, are possibly pregnant, or are lactating.
18	Individuals who participated in other clinical studies in the previous 3 months.
19	Individuals who are or whose family member is an employee of a health food company.
20	Individuals judged inappropriate for this study by the principal investigator.

Table S3: Degree classification of local findings.

Item	Group	n	0 week	4 week	8 week	12 week	16 week
Swell of concha nasalis inferior mucosa	LH2171	93	2.3 ± 0.9	2.1 ± 0.7**	2.1 ± 0.9**	2.0 ± 0.8**	2.0 ± 0.7**
	Placebo	94	2.4 ± 0.9	2.2 ± 0.7*	2.2 ± 0.8*	2.0 ± 0.8**	2.1 ± 0.8**
Color of concha nasalis inferior mucosa	LH2171	93	2.7 ± 1.0	2.4 ± 1.0**	2.3 ± 1.0**	2.4 ± 1.0**	2.3 ± 1.1**
	Placebo	94	2.8 ± 1.1	2.4 ± 1.0**	2.5 ± 1.0**	2.5 ± 1.1**	2.4 ± 1.0**
Aqueous secretion	LH2171	93	1.8 ± 0.6	1.7 ± 0.7	1.5 ± 0.7**	1.7 ± 0.7	1.6 ± 0.7*
	Placebo	94	1.7 ± 0.6	1.7 ± 0.7	1.4 ± 0.6**	1.7 ± 0.6	1.6 ± 0.7
Character of nasal mucus	LH2171	93	2.7 ± 1.4	2.4 ± 1.5*	2.1 ± 1.4**	2.7 ± 1.5	2.3 ± 1.4*
	Placebo	94	2.6 ± 1.4	2.3 ± 1.4	2.1 ± 1.4*	2.8 ± 1.5	2.3 ± 1.4

The symptom score was rated by the physician using a 5-point scale: 0, no symptoms; 1, mild; 2, moderate; 3, severe; 4, most severe. Data are expressed as the mean ± standard deviation. *p < 0.05, **p < 0.01 compare to 0 week by the Wilcoxon signed rank test.

Table S4. POMS 2 scores indicating psychological state (T score).

Items	Group	n	0 week	8 week	16 week
Anger - Hostility	LH2171	93	44.5 ± 7.7	44.9 ± 6.3	45.6 ± 7.9
	Placebo	94	44.8 ± 7.2	46.3 ± 8.6*	45.6 ± 7.1
Confusion - Bewilderment	LH2171	93	47.1 ± 9.0	47.8 ± 8.8	47.4 ± 8.5
	Placebo	94	48.1 ± 9.0	48.2 ± 8.2	47.0 ± 7.7
Depression - Dejection	LH2171	93	46.4 ± 6.7	46.9 ± 7.6	47.0± 7.1
	Placebo	94	46.6 ± 7.2	47.2 ± 7.8	47.0 ± 7.0
Fatigue - Inertia	LH2171	93	46.9 ± 8.0	47.4 ± 8.4	46.0 ± 8.0
	Placebo	94	47.6 ± 8.9	47.1 ± 8.5	46.5 ± 9.2
Tension - Anxiety	LH2171	93	45.6 ± 7.7	47.9 ± 8.5**	46.8 ± 7.4
	Placebo	94	46.5 ± 8.5	48.6 ± 8.4**	47.6 ± 8.4*
Vigor - Activity	LH2171	93	50.5 ± 9.9	49.6 ± 10.3	48.7 ± 10.0**
	Placebo	94	51.2 ± 8.3	49.8 ± 8.4*	50.6 ± 8.1
Friendliness	LH2171	93	51.5 ± 10.2	50.4 ± 10.6	50.0 ± 10.6*
	Placebo	94	51.4 ± 7.9	51.2 ± 9.0	50.8 ± 8.0
Total Mood Disturbance	LH2171	93	45.8 ± 8.1	46.8 ± 7.9*	46.7 ± 7.8
	Placebo	94	46.2 ± 7.7	47.3 ± 7.8	46.4 ± 7.4

Mean ± standard deviation. *p < 0.05, **p < 0.01 compared to 0 week by the Wilcoxon signed rank test.
